# Opioid Treatment Programs’ Medicaid Patient Retention Rates

**DOI:** 10.1001/jamanetworkopen.2025.53538

**Published:** 2026-01-23

**Authors:** Dylan E. DeLisle, Tami L. Mark, Chelsea Katz, William N. Dowd, Daniel Barch, Marianne Kluckman

**Affiliations:** 1 RTI International, Durham, North Carolina

## Abstract

**Importance:**

Retention on methadone and buprenorphine has been shown to be associated with reduced overdose and death. Opioid treatment programs (OTPs) are the only health care facilities in the US licensed to dispense methadone for opioid use disorder (OUD), and they increasingly prescribe buprenorphine. By measuring and comparing their patient retention rates, after adjusting for patient characteristics, OTPs can determine if their retention rate is low relative to other OTPs, which may motivate efforts to improve patient engagement and outcomes.

**Objective:**

To develop a standardized case mix–adjusted measure for evaluating Medicaid patient retention in OTPs.

**Design, Setting, and Participants:**

This cohort study used Medicaid outpatient claims and demographic data from 2018 to 2023. All OTPs in the US with at least 11 non–dually eligible Medicaid beneficiaries aged 18 years or older with an OUD diagnosis were included. Analyses were completed between January and October 2025.

**Exposure:**

OUD treatment at OTPs.

**Main Outcomes and Measures:**

The percentage of an OTP’s Medicaid patients who remained in treatment at the OTP for greater than 30 days, greater than 90 days, and greater than 180 days. Retention rates were calculated for 5 measurement years (2019 through 2023; eg, July 1, 2018, to June 30, 2019) and underwent case mix adjustment using Medicaid data and patient characteristics.

**Results:**

This study included 261 025 Medicaid beneficiaries (140 718 males [53.9%]; mean [SD] age, 39 [9.92] years) with 432 918 episodes of treatment at 1138 OTPs in measurement year 2023. The median (IQR) 30-day, 90-day, and 180-day retention rates were 61.2% (40.9%-73.5%), 41.5% (22.2%-54.8%), and 27.5% (11.4%-40.5%), respectively, in measurement year 2023.

**Conclusions and Relevance:**

This cohort study found low retention rates and variable treatment duration across OTPs. This result indicates opportunities for quality improvement.

## Key Points

**Question:**

What are the Medicaid patient retention rates in opioid treatment programs (OTPs) in the US?

**Findings:**

In this cohort study involving 261 025 Medicaid beneficiaries, the median OTP had a 30-day, 90-day, and 180-day retention rates of 61.2%, 41,5%, and 27.5%, respectively. The 30-day retention rates varied widely across OTPs.

**Meaning:**

The low and highly variable retention rates found in this study highlight an opportunity for OTPs to improve patent engagement and outcomes.

## Teaser

his cohort study assesses 30-, 90-, and 180-day retention rates among Medicaid beneficiaries participating in outpatient opioid treatment programs.

## Introduction

In 2024, 54 743 individuals died of an opioid overdose in the US.^1^ Opioid agonist medications, buprenorphine and methadone, are the primary treatments for opioid use disorder (OUD) and can substantially reduce the risk of overdose death.^2^ In the US, opioid treatment programs (OTPs) are the main source of these medications, are the only programs licensed to dispense methadone for OUD, and increasingly provide buprenorphine for OUD. Therefore, ensuring that OTPs deliver high-quality treatment is critical.

One marker of good OTP treatment quality is whether they retain patients in treatment and on OUD medications. Longer treatment duration with OUD medications is associated with lower risk of overdose and death.^3,4^ The American Society of Addiction Medicine recommends that individuals with OUD continue receiving methadone for at least 1 year, as early discontinuation within the first months is particularly dangerous.^5^ Previous studies found wide variation in OTP treatment retention rates. However, these studies evaluated data for a limited number of OTPs (ie, fewer than 30), and the most recent study used data from 2009 through 2011.^6,7,8,9,10,11^

This study aimed to develop a standardized case mix–adjusted measure for evaluating Medicaid patient retention in OTPs. Facility-level quality or performance measures (also known as audit and feedback) allow clinicians and practices to see how they are performing relative to their peers. The implicit idea is that when they see that their performance deviates from that of similar practitioners, they will be motivated to improve.^12^ A systematic review of more than 140 randomized clinical trials found that audit and feedback can be effective in improving practitioner performance.^13^ Developing and adopting standardized case mix–adjusted OTP retention measures would enable OTPs to compare their patient retention rates and may motivate efforts to increase patient engagement and enhance outcomes.

## Methods

### Data and Sample

In this cohort study, we used claims and enrollment data from the Centers for Medicare & Medicaid Services (CMS) Transformed Medicaid Statistical Information System Analytic Files (TAF) for the period from June 1, 2018, to December 31, 2023. Data analysis was completed between January and October 2025. The TAF captures enrollment and claims information from all Medicaid beneficiaries.^14^ We developed the OTP retention measure using Medicaid data because Medicaid beneficiaries composed more than half of all admissions to OTPs and approximately half of all opioid overdose deaths and because, unlike for privately insured individuals, data for all Medicaid beneficiaries were available.^15,16^ The Brany Institutional Review Board approved this study and waived the informed consent requirement under exemption 2(i)(a) and 4(ii) of the Common Rule (45 CFR § 46.104). We followed the Strengthening the Reporting of Observational Studies in Epidemiology (STROBE) reporting guideline.

The study population consisted of Medicaid beneficiaries, aged 18 years or older, who were not dually enrolled in Medicare and had a primary OUD diagnosis (*International Statistical Classification of Diseases and Related Health Problems, Tenth Revision* code F11.xx) on an OTP outpatient Medicaid claim. Beneficiaries dually eligible for Medicare were excluded because Medicare is the primary payer and the source of prescription drug coverage; therefore, the beneficiaries’ use of services were not captured in the Medicaid claims data.

To identify OTPs in the TAF, we used National Provider Identifier (NPI) numbers, which are unique, 10-digit numbers assigned to individual and organization health care providers. We determined whether the NPI numbers were for OTPs by using 3 sources: (1) NPIs on Medicaid claims for dispensed methadone for OUD; (2) the Substance Abuse and Mental Health Services Administration (SAMHSA) list of OTPs, which was linked to their NPI using the National Plan and Provider Enumeration System information and the OTP’s name and address; and (3) a CMS list of NPIs for OTPs that participate in Medicare. OTPs from New York and Illinois were excluded because their Medicaid claims omitted the procedure codes needed to identify dispensed medication for OUD.

### Measures

#### OTP Treatment Retention Rate

First, we determined the duration of treatment at an OTP for each patient-episode by calculating the number of days from treatment initiation to discontinuation at the same OTP. Initiation was defined as a visit to the OTP with no prior visits to the same OTP within the past 30 days. Discontinuation was determined if the patient had not received services from that OTP within 15 days or more. Services included outpatient visits and dispensed medications.

Second, we determined the percentage of each OTP’s patient-episodes that lasted greater than 30 days, greater than 90 days, or greater than 180 days. Only patients who were continuously enrolled in Medicaid over the duration of the follow-up period were included in each retention measure (30, 90, or 180 days). To protect patient privacy, measure results were reported only for OTPs with a total of 11 or more episodes in the year.

#### Measurement Year

OTP retention rates were calculated for 5 measurement years: 2019, 2020, 2021, 2022, and 2023. To be included in the denominator of a measurement year, an individual had to initiate an episode of treatment at the OTP between July 1 of the year prior to the measurement year and June 30 of the measurement year. For example, the 2023 measurement year included all episodes with initiation dates between July 1, 2022, and June 30, 2023. The duration of treatment was measured at least 180 days after treatment initiation. For example, episodes that began on June 30, 2023, were followed through December 31, 2023, to determine if the episode lasted greater than 180 days.

#### Case Mix Adjustment

Case mix adjustment of the retention rates was done to account for the fact that some OTPs treat patients with more severe conditions. Case mix adjustment creates a level playing field, so to speak, for comparing OTP retention rates. Variables for the case mix adjustment model were selected based on patient characteristics previously shown to be associated with retention and created using the TAF data.^17^ These variables included age, sex, methadone receipt 60 days prior to treatment initiation, buprenorphine receipt 60 days prior to treatment initiation, alcohol use disorder diagnosis 60 days prior to treatment initiation, drug use disorder diagnosis other than OUD 60 days prior to treatment initiation, mental health condition diagnosis 60 days prior to treatment initiation, any non–substance use disorder (SUD) reason for hospitalization 60 days prior to treatment initiation, SUD hospitalization 60 days prior to treatment initiation, and the Charlson Comorbidity Index (CCI). The CCI is calculated based on whether individuals have conditions shown to have a higher risk of mortality (ie, diabetes with diabetic complications, congestive heart failure, peripheral vascular disease, chronic pulmonary disease, liver disease, hemiplegia, kidney disease, leukemia, lymphoma, metastatic tumors, and AIDS).^18^ CCI ranges from 0 to 24, with 0 indicating the lowest risk of mortality and 24 indicating the highest risk of mortality.

To perform a case mix adjustment of the retention measure, we calculated each OTP’s unadjusted retention rate as a proportion: the number of episodes resulting in retention for greater than 30, greater than 90, or greater than 180 days following the initiation date divided by the total number of episodes for that clinic. We then applied case mix adjustment to these retention measures using the following equation: *retention_adj,p_* = *retention_unadj,p_* / *retention_exp,p_* × *retention_ovr_* (1), where *retention_unadj,p_* is the unadjusted retention rate for the OTP, *retention_exp,p_* is the expected retention rate for the OTP, and *retention_ovr_* is the overall retention rate across all episodes at all OTPs. The expected retention rate, *retention_exp,p_*, was computed as the arithmetic mean of predicted probabilities of retention across all episodes for the OTP using a logistic regression model.

### Statistical Analysis

Following standard approaches for testing quality measures, we assessed the reliability of the retention measure to establish whether the measure can meaningfully distinguish between OTPs that successfully retained a substantial proportion of their patients and OTPs that underperformed. The tests included Adams ρ, which is the ratio of between-OTP retention variance to the sum of the between-OTP and within-OTP variance for each OTP.^19^ Values of ρ greater than 0.7 represented acceptable reliability, and values of ρ greater than 0.9 represented excellent reliability. We also conducted a Kruskal-Wallis test to assess whether successful retention was randomly distributed at the episode level or whether differences existed between OTPs. We performed a Kolmogorov-Smirnov goodness-of-fit test to compare the observed distribution of OTP-level scores with a simulated distribution, assuming no underlying differences in the likelihood of retention between OTPs.

Two-sided *P* < .01 indicated statistical significance. Data analysis was performed with R, version 4.5 (R Core Team).

## Results

### Characteristics of Medicaid Patients Treated at OTPs 

In measurement year 2023, 261 025 patients were included in the retention measures. Table 1 provides a description of this patient population, which included 140 718 males (53.9%) and 120 307 females (46.1%) with a mean (SD) age of 39 (9.92) years. Most of these patients (232 628 [89.1%]) were defined by Medicaid as nondisabled.

**Table 1.** Characteristics of Patients Included in 30-Day Retention Analysis, 2023

Characteristic	Patients, No. (%) (N = 261 025)
Age range, y	
18-24	11 303 (4.3)
25-34	85 790 (32.9)
35-44	96 960 (37.2)
45-54	42 389 (16.2)
55-64	23 706 (9.1)
≥65	877 (0.3)
Sex	
Male	140 718 (53.9)
Female	120 307 (46.1)
Disability status	
Nondisabled	232 628 (89.1)
Disabled	28 397 (10.94)

The 261 025 patients included in measurement year 2023 had 432 918 treatment episodes, with a mean (SD) of 1.66 [1.65] episodes per patient. Table 2 shows the characteristics of each episode for measurement year 2023, and eTable 1 and eTable 2 in Supplement 1 provide the episode and patient characteristics, respectively, for the other measurement years. Among the 432 918 episodes used in the 30-day retention measure for measurement year 2023, 112 736 (26.0%) had dispensed methadone 60 days prior to treatment initiation, and 104 109 (24.0%) had dispensed buprenorphine or patient-filled buprenorphine prescription 60 days prior to treatment initiation. In 29 300 episodes (6.8%), alcohol use disorder was diagnosed 60 days prior to treatment initiation, and in 92 783 episodes (21.4%), drug use disorder other than OUD was diagnosed 60 days prior to treatment initiation. Additionally, in 116 170 episodes (26.8%), mental health disorder was diagnosed 60 days prior to OTP treatment initiation. There were 30 474 episodes (7.0%) of hospitalization for SUD in the 60 days prior to initiation, along with 7535 episodes (1.7%) of hospitalization for a reason other than SUD.

**Table 2.** Episode Characteristics by Retention Period, 2023

Characteristic	Episodes, No. (%)		
	30 d	90 d	180 d
Total episodes	432 918 (100)	427 350 (100)	415 298 (100)
CCI, mean (SD)^a^	0.14 (0.5)	0.14 (0.5)	0.14 (0.5)
Methadone receipt 60 d prior to treatment initiation	112 736 (26.0)	111 519 (26.1)	108 875 (26.2)
Buprenorphine receipt 60 d prior to treatment initiation	104 109 (24.0)	102 778 (24.1)	99 689 (24.0)
AUD diagnosis 60 d prior to treatment initiation	29 300 (6.8)	28 932 (6.8)	28 098 (6.8)
Drug use disorder diagnosis other than OUD diagnosis 60 d prior to treatment initiation	92 783 (21.4)	91 622 (21.4)	89 035 (21.4)
Mental health disorder diagnosis 60 d prior to treatment initiation	116 170 (26.8)	114 895 (26.9)	112 053 (27.0)
SUD hospitalization 60 d prior to treatment initiation	30 474 (7.0)	30 016 (7.0)	29 170 (7.0)
Hospitalization for reasons other than SUD 60 d prior to treatment initiation	7535 (1.7)	7365 (1.7)	7096 (1.7)

Abbreviations: AUD, alcohol use disorder; CCI, Charlson Comorbidity Index; OUD, opioid use disorder; SUD, substance use disorder.

^a^ CCI ranges from 0 to 24, with 0 indicating the lowest risk of mortality and 24 indicating the highest risk of mortality.

### Case Mix–Adjusted Results

The associations of the case mix logistic model’s independent variables with retention at an OTP are shown in Table 3. In measurement year 2023, older age, higher CCI, and hospitalization for reasons other than SUD in the 60 days prior to initiation were positively associated with 30-day retention. Receipt of methadone, receipt of buprenorphine, drug use disorder diagnosis other than OUD, and mental health disorder diagnosis in the 60 days prior to initiation were negatively associated with 30-day retention. Case mix adjustment resulted in 22 (1.9%) of the 1138 OTPs moving from the top quartile of 30-day retention to the middle 50th percentile and 9 (0.8%) of OTPs moving from the bottom quartile to the middle 50th percentile (eTable 3 in Supplement 1).

**Table 3.** Case Mix Variable Coefficients, 2023

Variable	Retention period					
	30 d		90 d		180 d	
	β-coefficient (SE)	*P* value	β-c oefficient (SE)	*P* value	β-c oefficient (SE)	*P* value
Intercept	−0.39 (0.01)	<.001	−1.23 (0.02)	<.001	−1.88 (0.02)	<.001
Female sex	0.01 (0.01)	.34	0.06 (0.02)	<.001	0.09 (0.01)	<.001
Aged 26-49 y	0.27 (0.01)	<.001	0.39 (0.02)	<.001	0.46 (0.02)	<.001
Aged ≥50 y	0.43 (0.02)	<.001	0.64 (0.02)	<.001	0.75 (0.02)	<.001
CCI^a^	0.02 (0.01)	.001	0.05 (0.01)	<.001	0.06 (0.01)	<.001
Methadone receipt 60 d prior to treatment initiation	−0.10 (0.01)	<.001	0.07 (0.01)	<.001	0.18 (0.01)	<.001
Buprenorphine receipt 60 d prior to treatment initiation	−0.76 (0.01)	<.001	−0.76 (0.01)	<.001	−0.78 (0.01)	<.001
AUD diagnosis 60 d prior to treatment initiation	0.02 (0.01)	.10	−0.13 (0.02)	<.001	−0.23 (0.02)	<.001
Drug use disorder diagnosis other than OUD 60 d prior to treatment initiation	−0.04 (0.01)	<.001	−0.22 (0.01)	<.001	−0.32 (0.01)	<.001
Mental health disorder diagnosis 60 d prior to treatment initiation	−0.07 (0.01)	<.001	−0.09 (0.01)	<.001	−0.09 (0.01)	<.001
SUD hospitalization 60 d prior to treatment initiation	0.02 (0.01)	.10	−0.04 (0.02)	.03	−0.05 (0.01)	.02
Hospitalization for reasons other than SUD 60 d prior to treatment initiation	0.08 (0.03)	.001	0.04 (0.03)	.16	0.01 (0.03)	.83

Abbreviations: AUD, alcohol use disorder; CCI, Charlson Comorbidity Index; OUD, opioid use disorder; SUD, substance use disorder.

^a^ CCI ranges from 0 to 24, with 0 indicating the lowest risk of mortality and 24 indicating the highest risk of mortality.

### Retention Rates Across OTPs 

Table 4 provides the 30-day, 90-day, and 180-day retention rates for the 5 measurement years 2019 through 2023. For the 2019 measurement year, there were 897 to 891 OTPs and 354 913 to 329 135 episodes included in the retention rates. There were 1138 to 1134 OTPs and 432 918 to 415 298 episodes for the 2023 measurement year. OTPs from every state except Wyoming, New York, and Illinois were captured. There were no SAMHSA-certified OTPs located in Wyoming. New York and Illinois were excluded due to missing procedure codes for OUD medications. OTPs had a mean (SD) number of 380 (930.63) newly initiating non–dually eligible eligible Medicaid episodes in measurement year 2023, with a median (IQR) value of 154 (68-338) care-initiating episodes.

**Table 4.** Thirty-Day, 90-Day, and 180-Day Retention Rates, 2019-2023

Measurement year	Retention period	No. of OTPs^a^	No. of episodes^a^	Mean retention rate (SD)	Median retention rate (IQR)
2019: July 1, 2018, to June 30, 2019	30 d	897	354 913	55.6 (29.2)	
					64.4 (33.3-79.2)
	90 d	895	345 045	41.4 (26.8)	46.0 (16.6-63.2)
	180 d	891	329 135	31.8 (23.5)	32.5 (9.1-51.1)
2020: July 1, 2019, to June 30, 2020	30 d	962	361 436	57.4 (27.1)	65.0 (37.9-78.8)
	90 d	960	354 662	42.2 (25.6)	45.5 (18.7-63.3)
	180 d	959	347 053	31.4 (22.8)	32.0 (9.9-48.5)
2021: July 1, 2020, to June 30, 2021	30 d	1034	400 398	57.8 (26.5)	65.1 (39.2-78.8)
	90 d	1034	397 936	42.5 (24.5)	46.6 (22.2-61.7)
	180 d	1034	393 757	31.4 (21.4)	32.1 (12.1-47.9)
2022: July 1, 2021, to June 30, 2022	30 d	1111	429 515	56.8 (25.3)	62.5 (39.2-76.7)
	90 d	1111	426 935	41.2 (23.2)	44.1 (23.5-58.3)
	180 d	1109	422 618	30.3 (20.0)	31.7 (13.2-44.6)
2023: July 1, 2022, to June 30, 2023	30 d	1138	432 918	55.3 (24.7)	61.2 (40.9-73.5)
	90 d	1136	427 350	38.9 (22.0)	41.5 (22.2-54.8)
	180 d	1134	415 298	27.4 (18.8)	27.5 (11.4-40.5)

Abbreviation: OTP, opioid treatment program.

^a^ In every denominator period, there were OTPs that met the 30-day retention denominator criteria but did not meet 90-day or 180-day retention denominator criteria, because patients did not meet the continuous enrollment criteria. Because patients were less likely to have continuous enrollment of more than 180 days rather than more than 30 days, the number of episodes was lower for the 180-day retention periods than the 30-day retention periods.

The median (IQR) 30-day, 90-day, and 180-day retention rates in the 2019 measurement year were 64.4% (33.3%-79.2%), 46.0% (16.6%-63.2%), and 32.5% (9.1%-51.1%), respectively (Table 4). In measurement year 2023, the median (IQR) 30-day, 90-day, and 180-day retention rates were 61.2% (40.9%-73.5%), 41.5% (22.2%-54.8%), and 27.5% (11.4%-40.5%), respectively. Across all the measures and years, the highest rate was the 30-day 65.1% (39.2%-78.8%) retention rate in measurement year 2021.

### Reliability Assessment

In all measurement years and for all follow-up durations, the tests of reliability indicated that the retention measure meaningfully distinguished between high-performing and underperforming OTPs. Mean (SD) ρ ranged from 0.95 (0.06) to 0.98 (0.03), and median (IQR) ρ ranged from 0.97 (0.93-0.99) to 0.99 (0.98-1.00). We rejected the null hypothesis of no OTP-level differences in retention under both the Kruskal-Wallis test (H statistic ranged from 72 111 to 105 087; all *P* < .001) and the Kolmogorov-Smirnov goodness-of-fit test (D statistic ranged from 0.53 to 0.59; all *P* < .001) for all years and follow-up durations.

## Discussion

This study found that there is considerable room for improvement in OTP retention rates. The median 30-day retention rate ranged from 61.2% to 65.1% across the 5 measurement years. Thus, in approximately half of the OTPs, one-third of the patients left their clinic within 30 days. Given recommendations that a minimum of 12 months is needed to fully benefit from methadone maintenance treatment and the elevated risk of overdose and death following discontinuation, efforts to increase retention in OTPs are needed.

Not only is the typical OTP retention rate low, but the study results also revealed sizable OTP-level differences in retention rates. Moreover, reliability testing indicated that this variation can be attributed to the OTP and not to random variation. Taken together, these statistics show that a standardized case mix–adjusted retention measure could be a valuable tool for OTPs to understand and increase their retention rates. Evidence-based approaches to improving retention and outcomes from OUD treatment include providing prompt and adequate dosage of methadone or buprenorphine to address craving and withdrawal, minimizing travel time to the clinic, and reducing stigma associated with treatment, among other strategies.^17,20,21,22^

The development of OTP quality measures has been a long-standing aim. For example, in 1995, RTI International and the National Association of State Alcohol and Drug Agency Directors conducted a National Institute on Drug Abuse–funded study to develop a methadone quality measurement system called Methadone Treatment Quality Assurance System.^23,24^ The project identified in-treatment outcome measures, such as drug use, retention in treatment, and patient satisfaction with treatment. However, the measures were never adopted. It is not clear whether pushback from clinicians and facilities, concerns about the validity of the measures, lack of resources, or other factors stymied the adoption of these measures. More recently, Dowd et al^24^ and Mark et al^25^ developed claims-based quality measures of retention in treatment for all substance use programs as part of the Shatterproof ATLAS quality measurement system used in several states. However, these measures were also not sustained. Sustainability of OTP measures will likely depend on a commitment from the federal and state governments. CMS currently provides public-facing quality metrics for a variety of health care facility types, including dialysis facilities, nursing homes, home health programs, and hospitals. CMS could feasibly implement the OTP retention measure we described using Medicaid and Medicare claims data. Additionally, states could create and implement this measure using their own data.

### Limitations

This study has some limitations that should be kept in mind. First, there is currently no standard approach to attributing patients to OTPs for quality measurement. In this study, we attributed any patient to the OTP if they had at least 1 visit to that OTP for a primary OUD diagnosis. The implicit assumption is that every patient who starts treatment at the OTP should ideally continue at the same OTP for 30, 90, or 180 days. However, there may be good reasons for a patient to leave treatment; for example, patients who are traveling out of town may only be “guest dosing” at the OTP. An alternative approach to attribution could have been to require the patient to make at least 2 or 3 visits to the OTP to be included in the retention measure. This approach would exclude patients who should have been retained at the OTP but left because of the inadequate care they received. Neither patient attribution approach is appropriate, and both have limitations.

Second, we made assumptions regarding the period for considering a new episode at an OTP (ie, 30 days with no prior visits to that OTP) and the period for considering discontinuation at the OTP (ie, 15 days with no services received from that OTP). These assumptions were based on what was used in past quality measures, such as the quality measures initiation and engagement in SUD treatment as well as feedback from an advisory panel consisting of patients and health care practitioners; however, one could argue that the period cut points should be longer or shorter. Ultimately, organizations that decide to use these quality measures could tailor their assumptions to better align with their goals and practices.

Third, our quality measure captured retention only at the OTP where treatment was initiated. In another research study, we are also developing measures of retention that cross OTPs. These measures will assess the proportion of an OTP's patient-episodes that had a transition to a medication for OUD when a patient left the OTP and the proportion of the OTP’s patient-episodes that continued OUD medication for at least 6 months regardless of whether a patient left the OTP.

Fourth, our study included Medicaid beneficiaries only. However, an estimated 75% of patients treated at OTPs have Medicaid coverage.^15^

## Conclusions

In this cohort study, the variation in treatment duration across OTPs indicates opportunities for quality improvement. Although retention in OUD treatment is critical for addressing the opioid crisis, there are currently no OTP-level measures to assess OTP treatment retention rates. The standardized case mix–adjusted retention measure developed in this study could facilitate identification of high- and low-performing OTPs and targeting of quality improvement efforts. Ideally, this measure would be adopted by federal or state agencies to share with OTPs and thus help increase OUD treatment retention and outcomes.

## Notes

Supplement 1. **eTable 1.** Episode Characteristics in the 60 Days Prior to Opioid Treatment Program Initiation Episode, by Measurement Year **eTable 2.** Demographic Characteristics by Denominator Initiation Claims and Unique Patient Identifiers, 2019-2023 **eTable 3.** Change in Number and Percent of OTPs in Top, Middle and Bottom Quartile After Casemix Adjustment

Supplement 2.
Data Sharing Statement
