# Supplementary material for: Opioid Treatment Programs’ Medicaid Patient Retention Rates
Source: JAMA Netw Open. 2026 Jan 23;9(1):e2553538. doi: 10.1001/jamanetworkopen.2025.53538 (PMC12831151; doi:10.1001/jamanetworkopen.2025.53538)
Supplement: Supplement 1. — eTable 1. Episode Characteristics in the 60 Days Prior to Opioid Treatment Program Initiation Episode, by Measurement Year eTable 2. Demographic Characteristics by Denominator Initiation Claims and Unique Patient Identifiers, 2019-2023 eTable 3. Change in Number and Percent of OTPs in Top, Middle and Bottom Quartile After Casemix Adjustment [file jamanetwopen-e2553538-s001.pdf]

Supplemental Online Content

DeLisle DE, Mark TL, Katz C, Dowd WN, Barch D, Kluckman M. Opioid treatment programs' Medicaid patient retention rates. *JAMA Netw Open*. 2026;9(1):e2553538. doi:10.1001/jamanetworkopen.2025.53538

eTable 1. Episode Characteristics in the 60 Days Prior to Opioid Treatment Program Initiation Episode, by Measurement Year

eTable 2. Demographic Characteristics by Denominator Initiation Claims and Unique Patient Identifiers, 2019-2023

eTable 3. Change in Number and Percent of OTPs in Top, Middle and Bottom Quartile After Casemix Adjustment

This supplemental material has been provided by the authors to give readers additional information about their work.

© 2026 DeLisle DE et al. *JAMA Network Open*

eTable 1. Episode Characteristics in the 60 days prior to Opioid Treatment Program Initiation Episode, by Measurement Year

		Retention Period		
**Episode Characteristic**	**Measurement Year**	**30-Day No. Episodes (%)**	**90-Day No. Episodes (%)**	**180-Day No. Episodes (%)**
Total Denominator Eligible Episodes				
	2019	354913 (100)	345045 (100)	329135 (100)
	2020	361436 (100)	354662 (100)	347053 (100)
	2021	400398 (100)	397936 (100)	393757 (100)
	2022	429515 (100)	426935 (100)	422618 (100)
	2023	432918 (100)	427350 (100)	415298 (100)
Charlson Comorbidity Index, mean (SD)				
	2019	0.16 (0.53)	0.16 (0.50)	0.16 (0.50)
	2020	0.16 (0.49)	0.15 (0.49)	0.15 (0.48)
	2021	0.15 (0.48)	0.14 (0.47)	0.14 (0.47)
	2022	0.14 (0.48)	0.14 (0.47)	0.14 (0.47)
	2023	0.14 (0.48)	0.14 (0.48)	0.14 (0.47)
Received methadone 60 days prior to treatment initiation				
	2019	102388 (28.8)	100342 (29.1)	96904 (29.4)
	2020	97593 (27.0)	96286 (27.1)	94768 (27.3)
	2021	118629 (29.6)	118124 (29.7)	117163 (29.8)
	2022	121700 (28.3)	121100 (28.4)	120095 (28.4)
	2023	112736 (26.0)	11519 (26.1)	108875 (26.2)
Received buprenorphine 60 days prior to treatment Initiation				
	2019	59622 (16.8)	58048 (16.8)	55554 (16.9)
	2020	66690 (18.5)	65600 (18.5)	64286 (18.5)
	2021	79813 (19.9)	79342 (19.9)	78507 (19.9)
	2022	93619 (21.8)	93105 (21.8)	92209 (21.8)
	2023	104109 (24.0)	102778 (24.1)	99689 (24.0)
		**Retention Period**		

© 2026 DeLisle DE et al. *JAMA Network Open*

Episode Characteristic	Measurement Year	30-Day No. Episodes (%)	90-Day No. Episodes (%)	180-Day No. Episodes (%)
Diagnosed with Alcohol Use Disorder 60 days prior to treatment initiation				
	2019	18525 (5.2)	18034 (5.2)	17228 (5.2)
	2020	21953 (6.1)	21560 (6.1)	21049 (6.1)
	2021	24897 (6.2)	24684 (6.2)	24279 (6.2)
	2022	24618 (5.7)	24416 (5.7)	24056 (5.7)
	2023	29300 (6.8)	28932 (6.8)	28098 (6.8)
Diagnosed with Drug Use Disorder other than OUD 60 days prior to treatment initiation				
	2019	61189 (17.2)	59447 (17.2)	56452 (17.2)
	2020	67518 (18.7)	66258 (18.7)	64646 (18.6)
	2021	75903 (19.0)	75293 (18.9)	74259 (18.9)
	2022	83467 (19.4)	82807 (19.4)	81672 (19.3)
	2023	92783 (21.4)	91622 (21.4)	89035 (21.4)
Diagnosed with a Mental Health condition 60 days prior to treatment initiation				
	2019	88843 (25.0)	86748 (25.1)	83201 (25.3)
	2020	95129 (26.3)	93636 (26.4)	91726 (26.4)
	2021	111313 (27.8)	110567 (27.8)	109244 (27.7)
	2022	111674 (26.0)	110927 (26.0)	109630 (25.9)
	2023	116170 (26.8)	114895 (26.9)	112053 (27.0)
Hospitalized for a substance use disorder 60 days prior to treatment initiation				
	2019	24638 (6.9)	23868 (6.9)	22651 (6.9)
	2020	26526 (7.3)	25914 (7.3)	25215 (7.3)
	2021	28933 (7.2)	28617 (7.2)	28110 (7.1)
	2022	29880 (7.0)	29524 (6.9)	28970 (6.9)
	2023	30474 (7.0)	30016 (7.0)	29170 (7.0)

© 2026 DeLisle DE et al. *JAMA Network Open*

		Retention Period		
**Episode Characteristic**	**Measurement Year**	**30-Day No. Episodes (%)**	**90-Day No. Episodes (%)**	**180-Day No. Episodes (%)**
Hospitalized for reasons other than substance use disorder 60 days prior to treatment initiation				
	2019	7523 (2.1)	7241 (2.1)	6856 (2.1)
	2020	7387 (2.0)	7164 (2.0)	6906 (2.0)
	2021	7525 (1.9)	7380 (1.9)	7175 (1.8)
	2022	7649 (1.8)	7452 (1.7)	7244 (1.7)
	2023	7535 (1.7)	7365 (1.7)	7096 (1.7)

© 2026 DeLisle DE et al. *JAMA Network Open*

eTable 2. Demographic Characteristics by Denominator Initiation Claims and Unique Patient Identifiers, 2019-2023

Patient Characteristic	2019 No. Patients (%)	2019 No. Episodes (%)	2020 No. Patients (%)	2020 No. Episodes (%)	2021 No. Patients (%)	2021 No. Episodes (%)	2022 No. Patients (%)	2022 No. Episodes (%)	2023 No. Patients (%)	2023 No. Episodes (%)
**Age**										
18-24	12574 (5.5)	19006 (5.4)	11429 (5.0)	17836 (4.9)	12072 (4.9)	19261 (4.8)	11812 (4.6)	18443 (4.3)	11303 (4.3)	17473 (4.0)
25-34	91444 (39.7)	142956 (40.3)	88481 (38.7)	142017 (39.3)	91712 (37.6)	153503 (38.3)	91184 (35.2)	152332 (35.5)	85790 (32.9)	143127 (33.1)
35-44	67384 (29.2)	105173 (29.6)	72367 (31.7)	115022 (31.8)	80492 (33.0)	132654 (33.1)	91824 (35.5)	153713 (35.8)	96960 (37.2)	162355 (37.5)
45-54	35123 (15.2)	52706 (14.9)	34952 (15.3)	54065 (15.0)	36722 (15.0)	58472 (14.6)	40397 (15.6)	66477 (15.5)	42389 (16.2)	69904 (16.2)
55-64	23496 (10.2)	34376 (9.7)	20799 (9.1)	31791 (8.8)	22540 (9.2)	35536 (8.9)	23061 (8.9)	37352 (8.7)	23706 (9.1)	38665 (8.9)
65+	508 (0.2)	696 (0.2)	479 (0.2)	705 (0.2)	619 (0.3)	972 (0.2)	763 (0.3)	1198 (0.3)	877 (0.3)	1394 (0.3)
**Sex**										
Male	119496 (51.8)	182597 (51.5)	119730 (52.4)	188532 (52.2)	130218 (53.3)	212937 (53.2)	138633 (53.5)	228422 (53.2)	140718 (53.9)	231996 (53.6)
Female	111033 (48.2)	172316 (48.6)	108777 (47.6)	172904 (47.8)	113939 (46.7)	187461 (46.8)	120408 (46.5)	201093 (46.8)	120307 (46.1)	200922 (46.4)
Disability Status										
Non-Disabled	196140 (85.1)	302940 (85.4)	198514 (86.9)	314147 (86.9)	213974 (87.6)	352987 (88.2)	229854 (88.7)	381784 (88.9)	232628 (89.1)	386312 (89.2)
Disabled	34389 (15.0)	51973 (14.6)	29993 (13.1)	47289 (13.1)	30183 (12.4)	47411 (11.8)	29187 (11.3)	47731 (11.1)	28397 (10.9)	46606 (10.8)

© 2026 DeLisle DE et al. *JAMA Network Open*

eTable 3. Change in Number and Percent of OTPs in Top, Middle and Bottom Quartile After Casemix Adjustment

Measure	N	Observed	Case-mix Adjusted		
			Top 25%	Middle 50%	Bottom 25%
30-day retention	1138	Top 25%	262 (23%)	22 (1.9%)	0 (0.0%)
30-day retention	1138	Middle 50%	22 (1.9%)	539 (47.4%)	9 (0.8%)
30-day retention	1138	Bottom 25%	0 (0.0%)	9 (0.8%)	275 (24.2%)
90-day retention	1136	Top 25%	265 (23.3%)	19 (1.7%)	0 (0.0%)
90-day retention	1136	Middle 50%	19 (1.7%)	537 (47.3%)	12 (1.1%)
90-day retention	1136	Bottom 25%	0 (0.0%)	12 (1.1%)	272 (23.9%)
180-day retention	1134	Top 25%	261 (23%)	22 (1.9%)	0 (0.0%)
180-day retention	1134	Middle 50%	22 (1.9%)	535 (47.2%)	11 (1%)
180-day retention	1134	Bottom 25%	0 (0.0%)	11 (1%)	272 (24%)

© 2026 DeLisle DE et al. *JAMA Network Open*
