# Supplementary material for: Opioid Treatment Programs’ Medicaid Patient Retention Rates
Source: JAMA Netw Open. 2026 Jan 23;9(1):e2553538. doi: 10.1001/jamanetworkopen.2025.53538 (PMC12831151; doi:10.1001/jamanetworkopen.2025.53538)
Supplement: Supplement 2. — Data Sharing Statement [file jamanetwopen-e2553538-s002.pdf]

Data Sharing Statement

DeLisle. Opioid Treatment Programs' Medicaid Patient Retention Rates. *JAMA Netw Open*. Published January 23, 2026. doi:10.1001/jamanetworkopen.2025.53538

Data: To conduct this study, the authors licensed the Transformed Medicaid Statistical Information System Analytic Files (TAF) data through the Centers for Medicare & Medicaid Services.
Data available: No

Additional Information: The TAF data dictionary is available at resdac.org.
Explanation for why data not available: Because the data are identifiable, they may not be shared.
